# Supplementary figures and images for: Comparative Proteomic Analyses of Poorly Motile Swamp Buffalo Spermatozoa Reveal Low Energy Metabolism and Deficiencies in Motility-Related Proteins
Source: Animals (Basel). 2022 Jul 1;12(13):1706. doi: 10.3390/ani12131706 (PMC9264820; doi:10.3390/ani12131706)

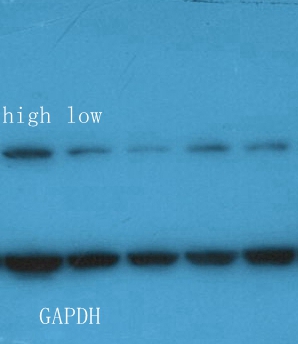

Supplement: Supplementary file 1 [file animals-12-01706-s001.zip › Supplementary figure/AKAP3 raw.jpg]

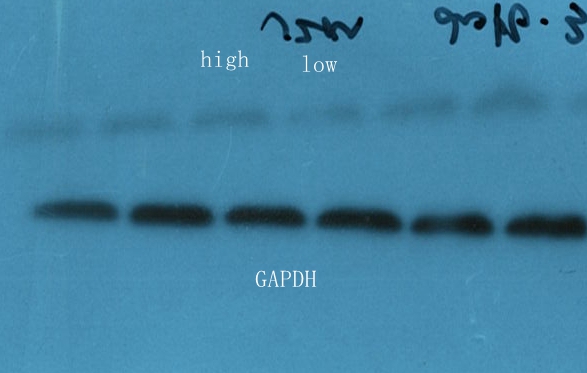

Supplement: Supplementary file 1 [file animals-12-01706-s001.zip › Supplementary figure/CCDC40 raw.jpg]

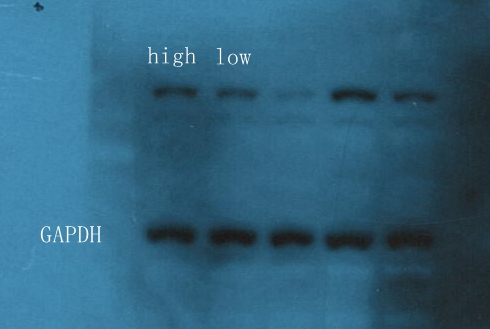

Supplement: Supplementary file 1 [file animals-12-01706-s001.zip › Supplementary figure/PRM1 raw.jpg]

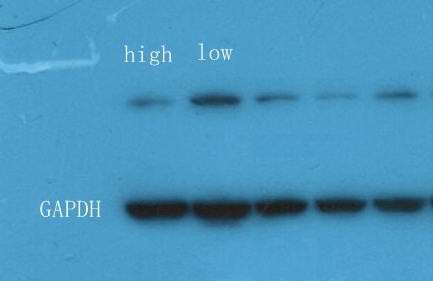

Supplement: Supplementary file 1 [file animals-12-01706-s001.zip › Supplementary figure/SDC2 raw.jpg]

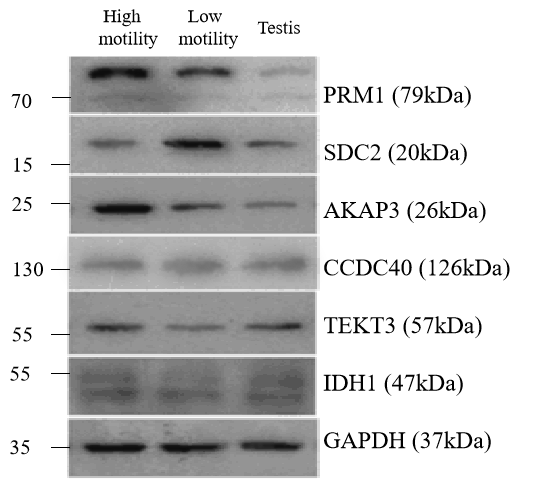

Supplement: Supplementary file 1 [file animals-12-01706-s001.zip › Supplementary figure/Supplementary Figure S1.png]

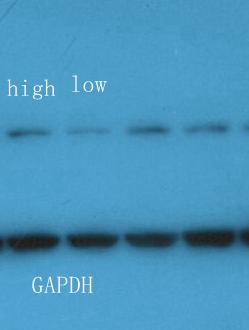

Supplement: Supplementary file 1 [file animals-12-01706-s001.zip › Supplementary figure/TEKT3 raw.jpg]
